# Nano–Bio Interactions: Nanomedicine and Nanotoxicology

**DOI:** 10.3390/ijerph15061222

**Published:** 2018-06-10

**Authors:** Hugh J. Byrne, Sourav P. Mukherjee, Pratap C. Naha

**Affiliations:** 1 FOCAS Research Institute, Dublin Institute of Technology, Kevin Street, Dublin D08 X622, Ireland; 2 Molecular Toxicology Unit, Institute of Environmental Medicine (IMM), Karolinska Institutet, 17177 Stockholm, Sweden; sourav.mukherjee@ki.se; 3 Department of Radiology, Perelman School of Medicine, University of Pennsylvania, Philadelphia, PA 19104, USA; Pratap.naha@uphs.upenn.edu

The 21st century has truly become the age of nanotechnology. Nanomaterials, design strategies, and processing have already made a significant impact in areas of materials science and electronics, with many commercial applications already being available on the consumer market. However, the ability to manipulate material functions and interactions on a scale of tens of nanometers, e.g., biological subcellular organelles, may yet prove to have the most significant impact on human health and the environment. The design of nanometer-scale contrasts, drug and nutrient delivery agents, as well as nanostructured materials for improved biocompatible interfaces, has opened up a whole new realm of nanomedicine. Equally, however, the ever increasing, to date largely unregulated, proliferation of nanoscale materials into the consumer environment has raised concerns over the potential detrimental impacts of uncontrolled exposure on human health and the environment.

This Special Issue of *International Journal of Environmental Research and Public Health* (*IJERPH*) collates articles describing the current state of play in understanding the fundamentals of nano–bio interactions, how they potentially impact human health and the environment, and how research has progressed our understanding of how they can be exploited for the betterment of healthcare. It comprises six articles, from thirteen institutions and seven countries across the world. It covers aspects of cytotoxicity, genotoxicity and ecotoxicity of a range of organic and inorganic nanoparticles, as well as aspects of toxicity monitoring and nanomedical applications.

Kangze Liu, et al. [1] investigated the toxicological effect of gold nanoparticles in fungi. In this study, gold nanoparticles with different sizes, i.e., 0.7 to 400 nm, and different shapes, i.e., star, spherical and flower were examined. Toxicity studies of these gold nanoparticles were carried out in three different fungi, i.e., *Aspergillus niger*, *Mucor hiemalis*, and *Penicillium chrysogenum*. It was found that larger and non-spherical gold nanoparticles are relatively more toxic than the smaller and spherical gold nanoparticle tested in this study. In addition, *A. niger* was the most sensitive to the gold nanoparticles, whereas *M. hiemalis* was found to be the least. David M. Metzler, et al. [2] reported the adverse effects of titanium dioxide nanoparticles to algae. This study shows that titanium dioxide nanoparticle generates free radicals, which inhibit the growth of algae. Merve Ozkaleli and Ayca Erdem [3] also reported on the adverse, inhibitory, effects of titanium dioxide nanoparticle exposure on the growth of algae. Interestingly, they found that different water conditions play a significant role in the toxicity. A soft synthetic freshwater system, pH 7.3 ± 0.2 was found to be the most effective water type for growth inhibition, whereby 95% algal cell death was recorded at 50, 100 and 500 mg/L nanoparticle concentrations.

Both fungi and algae are found in most terrestrial, marine, and freshwater environments and are key trophic components of the ecosystem. They may therefore be considered important sentinel species to monitor potential adverse effects of nanoparticle exposure to the environment. Notably, both gold and titanium dioxide nanoparticles are examples which are currently commercially available for both fundamental research, and indeed in consumer goods. The continued reports of adverse environmental effects are therefore of concern.

Zinc oxide nanoparticles are also already widely used in consumer products, notably for dermal applications. Pascal Ickrath et al. [4] investigated the cytotoxic and genotoxic effects of zinc oxide nanoparticle in human mesenchymal stem cells at low-dose concentrations after long-term and repetitive exposure. It was found that zinc oxide nanoparticles were internalized in the cells, and accumulated for up to six weeks. Cytotoxicity was observed at high doses, while genotoxic effects were observed at moderate and low doses. Repetitive exposure enhanced cytotoxic, but not genotoxic effects. 

Pratap C. Naha et al. [5] provided an up to date review of polyamidoamine (PAMAM) dendrimer toxicity. These macromolecular nanoparticulate materials are much vaunted for potential biomedical applications, due to their moleculary tailorable chemical structures and surface functionality. The physico–chemical properties of PAMAM dendrimers play an important role in their bio-interactions, such that structure activity relationships (SARs) can be established and they can serve as the basis for predictive toxicology models. Their toxicity is dependent on generation, and therefore number of surface of amino groups, and is significantly reduced when the surface amino groups are blocked via acylation or are modified to hydroxyl or carboxylic acid. A detailed understanding of their SARs can also guide strategies for targeted delivery in nanomedical applications. 

The work of Luisana Di Cristo, et al. [6] describes the development of an in vitro tool for assessing the efficacy of nanoparticulate inhalation therapies, supplied by aerosol. Measuring parameters such as hydrodynamic size distributions, cell viability, cell monolayer integrity, cell morphology and pro-inflammatory cytokines secretion of cell cultures at the air–liquid interface; they demonstrate an effective in vitro tool for assessing the correlation between the physico–chemical properties of nanoparticles and their biological behavior, when nanoparticles are used as drug delivery systems. 

This range of studies demonstrates that the field of research of environmental and health impacts of nanomaterials exposure is still vibrant, and causing concern. Notably, the increased research highlights environmental and health risks of prominent nanomaterials which are already commercially available and used extensively in consumer products, heightening awareness of the requirement for assessment of realistic exposure scenarios. The scope of research also highlights, however, the improved understanding of the nanoparticle/biological interactions, towards the development of nanomedical applications for improved human healthcare and ultimately quality of life.
